# Identification and Characterization of a Cleavage Site in the Proteolysis of Orf Virus 086 Protein

**DOI:** 10.3389/fmicb.2016.00538

**Published:** 2016-04-20

**Authors:** Xiaoping Wang, Bin Xiao, Jiafeng Zhang, Daxiang Chen, Wei Li, Ming Li, Wenbo Hao, Shuhong Luo

**Affiliations:** ^1^Institute of Antibody Engineering, School of Biotechnology, Southern Medical UniversityGuangzhou, China; ^2^Department of Laboratory, Medicine Nongken Centre, Hospital of GuangdongZhanjiang, China; ^3^Department of Laboratory Medicine, Guangzhou General Hospital of Guangzhou Military CommandGuangzhou, China; ^4^Guangdong Provincial Key Laboratory of Tropical Disease Research, Department of Epidemiology, School of Public Health, Southern Medical UniversityGuangzhou, China; ^5^State Key Laboratory of Organ Failure, Guangdong Provincial Key Laboratory of Tropical Disease Research, School of Biotechnology, Southern Medical UniversityGuangzhou, China

**Keywords:** orf virus, ORFV086, proteolysis, pulse-chase

## Abstract

The orf virus (ORFV) is among the parapoxvirus genus of the poxviridae family, but little is known about the proteolytic pathways of ORFV encoding proteins. By contrast, the proteolysis mechanism of the vaccinia virus (VV) has been extensively explored. Vaccinia virus core protein P4a undergoes a proteolytic process that takes place at a conserved cleavage site Ala-Gly-X (where X is any amino acid) and participates in virus assembly. Bioinformatics analysis revealed that an ORFV encoding protein, ORFV086, has a similar structure to the vaccinia virus P4a core protein. In this study, we focus on the kinetic analysis and proteolysis mechanism of ORFV086. We found, via kinetic analysis, that ORFV086 is a late gene that starts to express at 8 h post infection at mRNA level and 12–24 h post infection at the protein level. The ORFV086 precursor and a 21 kDa fragment can be observed in mature ORFV virions. The same bands were detected at only 3 h post infection, suggesting that both the ORFV086 precursor and the 21 kDa fragment are viral structural proteins. ORFV086 was cleaved from 12 to 24 h post infection. The cleavage took place at different sites, resulting in seven bands with differing molecular weights. Sequence alignment revealed that five putative cleavage sites were predicted at C-terminal and internal regions of ORFV086. To investigate whether those cleavage sites are involved in proteolytic processing, full length and several deletion mutant ORFV086 recombinant proteins were expressed and probed. The GGS site that produced a 21 kDa cleavage fragment was confirmed by identification of N/C-terminal FLAG epitope recombinant proteins, site-directed mutagenesis and pulse-chase analysis. Interestingly, chase results demonstrated that, at late times, ORFV086 is partially cleaved. Taken together, we concluded that GGS is a cleavage site in ORFV086 and produces a 21 kDa fragment post infection. Both ORFV086 precursor and the 21 kDa fragment are structural proteins of mature ORFV virions. ORFV086 and its cleaved products are indispensable for correct assembly of mature viral particles and this proteolytic processing of ORFV086 may play an essential role in viral morphogenic transition.

## Introduction

The orf virus (ORFV), the prototype member of the *Poxviridae Parapoxvirus* genus (Diel et al., 2011), is a double-stranded DNA virus. It is brick-shaped or oval and, under electron microscopy, has a “criss-cross” arrangement on its surface. The virus particle structure is complex and includes the core, the side body and envelope. The genome of the orf virus is 138kb and is rich in G+C content (64%) (Delhon et al., 2004). Both ends of the genome encode immunomodulatory proteins and are highly variable, while genes in the central region of the genome (ORF009-ORFV111) are highly conserved and play key roles in replication, assembly and viral release (Mercer et al., 1987, 2006; Cottone et al., 1998; Delhon et al., 2004). Genomic analysis also shows that the core region of the genome is very similar to that of vaccinia virus (VV) (Mercer et al., 2006). Currently, research regarding the replication, assembly, morphogenesis and immune mechanisms of the ORFV is scarce.

Analysis of different ORFV strains shows that the protein encoded by the ORFV086 gene is expressed in the core of the virus and has structural similarities with the VV core protein P4a and other poxvirus homologs (VanSlyke et al., 1991; Vanslyke et al., 1991; Heljasvaara et al., 2001). The P4a protein is the most abundant structural protein in the VV, accounting for 14% of the virion mass (Heljasvaara et al., 2001). Encoded by the A10L gene (Rodriguez et al., 2006), P4a is expressed at late times in the viral infection as a 102 kDa protein, which is subsequently processed into three polypeptides after proteolysis. The three polypeptides are 62, 23, and 9 kDa in size. This processing is important for maturation of the VV progeny (Vanslyke et al., 1991; Heljasvaara et al., 2001). Several structural core protein precursors of VV, such as P4a, P4b, and P25K, have a conserved cleavage motif, Ala-Gly-X (where X is any amino acid), and are catalyzed by a VV encoded proteinase (Byrd and Hruby, 2006). In the case of vaccinia virus, proteolysis of the core protein is characterized by: (1) having an AGX motif, (2) expression late in the infection, and (3) packaging into assembling virions composed of viral core particles (Byrd and Hruby, 2006; Yang, 2007). The core proteins of other DNA viruses, such as adenovirus and African swine fever virus, also undergo specific proteolysis in the processes of viral replication and morphogenesis (López-Otín et al., 1989). Differing from the AGX motif utilized by the VV core protein (Whitehead and Hruby, 1994), the proteolysis of the adenovirus core protein occurs at the Gly-Gly-X motif (López-Otín et al., 1989), as do three core proteins of African swine fever virus (López-Otín et al., 1989; Lee and Hruby, 1993). Proteolysis of structural proteins during viral replication is a common theme (Lee and Hruby, 1993) among many DNA viruses (T4 phage6, adenovirus, Hellen and Wimmer, 1992b) and RNA viruses (picornavirus, Hellen and Wimmer, 1992a, nodavirus, and retrovirus).

The ORFV086 gene of the NA1/11 strain is 2718 bp (Li H. et al., 2012), encoding the 100.05 kDa ORFV086 protein. It is a highly conserved gene located in the middle region of the genome, and plays a key role in viral replication and morphogenesis (Li W. et al., 2012). Bioinformatics analysis revealed that the ORFV086 protein is similar in structure to the VV precursor core protein P4a and other poxvirus homologs (Heljasvaara et al., 2001). Of particular interest is whether or not this ORFV086 protein is proteolytically processed similarly to the VV P4a core protein. Sequence alignments of the P4a protein of vaccinia virus Copenhagen strain (VACV P4a) and the ORFV086 protein of NA1/11 (ORFV086 NA1/11), based on their predicted amino acid sequences, found that the proteolytic sites of P4a are AGS and AGT, while the corresponding amino acid residues of the ORFV086 protein are GGA and GGS. However, this is similar to the proteolytic motif (Gly-Gly-X) of adenoviruses and the African swine fever virus core protein (López-Otín et al., 1989). Simultaneously, amino acid sequencing of the full-length ORFV086 protein was carried out to retrieve the AGX motif and found AGP (residues 180-182), AGK (413-415), and AGL (496-498). If the ORFV086 protein is processed similarly to the VV P4a protein, we hypothesized that these five sites (AGP, AGK, AGL, GGA, and GGS) are likely to be its proteolytic sites.

To test this hypothesis, it was necessary to study whether proteolysis of the ORFV086 protein occurs at these sites. In this study, to describe the proteolytic characteristics of the ORFV086 protein, the procedure outlined below was followed: (1) Kinetic analysis of the ORFV086 protein, (2) construction of the full length ORFV086 carrier and different sized ORFV086 fragments to study the proteolytic site location in ORFV086, and (3) determining the proteolytic site of ORFV086 protein by site-directed mutagenesis and pulse-chase analysis. From these results, we describe the proteolytic properties of ORFV086, laying the foundation for understanding the process of ORFV replication, assembly, and morphogenesis.

## Materials and methods

### Cells and virus

Primary ovine fetal turbinate (OFTu) cells were cultured at 37°C in MEM complemented with 10% FBS. The ORFV NA1/11 virus used in this experiment was isolated from a sheep diagnosed with orf in the Jilin province of China. NA1/11 grown in OFTu cells was purified by sucrose gradient ultracentrifugation as described previously (Joklik, 1962; Li H. et al., 2012).

### Sequence alignment and phylogenetic analysis of ORFV086 protein

Sequence alignment of P4a protein of VV strain Copenhagen (VACV P4a, GenBank ID: AGJ92402.1) with NA1/11 ORFV086 protein (ORFV086 NA1/11, GenBank ID: AHZ33783.1) and multiple alignments of corresponding ORFV086 proteins among orf virus strains (OV-IA82, GenBank ID: AAR98181.1, NZ2, GenBank ID: ABA00603.1, OV-SA00, GenBank ID: AAR98311.1, D1701, GenBank ID: ADY76725.1, B029, GenBank ID: AHH34275.1, NA1/11, GenBank ID: AHZ33783.1, HN3, GenBank ID: KU160637, FJ-GO, GenBank ID: AKU76707, FJ-NP, GenBank ID: AKU76839, FJ-SJ, GenBank ID: AKU76963, FJ-YX, GenBank ID: AKU76575) were created by using CLUSTAL W software based on the amino acid sequences. Different homologous sequences were shaded with different colors. MEGA 5.0 (Tamura et al., 2011) software was used to construct a phylogenetic tree of different orf virus strains, VACV and bovine papular stomatitis virus (BPSV) strain BV-AR02 using the Neighbor-Joining method (Saitou and Nei, 1987; Lojkic et al., 2010; Li W. et al., 2012; Chi et al., 2013).

### Construction and site-directed mutation of expression plasmids

In order to identify the cleavage sites of ORFV086 protein, we designed and constructed different ORFV086 fragment expression plasmids based on two putative sites (GGA, GGS). The primer design was based on published NA1/11 ORFV086 sequences (Accession Number: JQ729675.1.). Different ORFV086 fragments were amplified by PCR using PrimeStar DNA polymerase and cloned into eukaryotic expression vectors pCMV-Tag2B and pCMV-Tag4A, in which FLAG-epitopes (Tan et al., 2009) were appended to the N-terminus and C-terminus. Site-directed mutation of two putative sites (GGA, GGS) and three AGX (AGP, AGK, AGL) motifs of 4A-086 were performed using the Muta-direct™ Site-directed Mutagenesis Kit (SBS Genetech) to convert each of them into IDI. Directed Mutagenesis primers were as follows (mutation sites are underlined): site-directed mutation from AGP to IDI, 5′-ATC TACCCCAACATCATCGACATCGCCGAGATCGGCTTTC -3′, site-directed mutation from AGK to IDI, 5′- TTTCGGACGCGCTCGCGATCGATATAGAGCCCCTCCGCGTG -3′, site-directed mutation from AGL to IDI, 5′-GTG CTCATGGCCTACATCGACATCAGGATGGAAGAC AAG-3′, site-directed mutation from GGA to IDI, 5′- CGCGCATCATCTACATCGA CATCAAGGACGCCGCCGAG -3′, and site-directed mutation from GGS to IDI, 5′- CGTCTGGTGACCGTCATCGACATCCCCGATATCACGATC -3′. All constructed plasmids and mutants were sequenced for confirmation.

### Production of fusion protein and polyclonal antiserum

To produce a large quantity of ORFV086 proteins in *E. coli*, we constructed the prokaryotic recombinant plasmid pET33b-086, whose coding exactly matched the full-length ORFV086 protein. The pET33b-086 plasmid was transformed into *E. coli* BL21 (DE3) pLys and induced by IPTG at 37°C. The target protein was isolated by gel purification. Polyclonal antiserum against ORFV086 was produced using purified pET33b-086 as an immunogen. Briefly, purified protein was mixed with adjuvants in a ratio of 1:1 (v/v) and emulsified by passage through a 22-gauge needle. The mixtures were subsequently injected into two male New Zealand white rabbits in a low-dose multi-point injection from different channels. A total of 500 μg antigens were emulsified with an equal amount of Freund's complete adjuvant for the primary immunization, followed by emulsification with Freund's incomplete adjuvant for all subsequent immunizations. Small amounts of blood were separated 1 week after the third immunization to test titers by ELISA. Polyclonal antisera were collected by conventional methods (Xiao et al., 2016).

### Transient expression

OFTu cells, both infected and uninfected with ORFV NA1/11, were transfected with plasmids according to the Lipofectamine® 2000 Reagent protocol. Briefly, seed cells were 80–90% confluent at the time of transfection. For each well of a 6-well plate, plasmid DNA and lipid were mixed gently in 200 μl Opti-MEM media by adding 2.5 μg plasmid DNA and 5 μl Lipofectamine Reagent and incubated for 20 min at room temperature. Finally, the DNA-lipid complex was added to the infected and uninfected cells, which had been maintained in 800 μl Opti-MEM media prior to transfection. At 6 h after transfection, the transfection media was replaced with fresh MEM media complemented with 10% FBS and the cells were incubated for an additional 1–3 days at 37°C.

### Western blotting

Transfected or infected cells were lysed in 1x sample loading buffer and boiled for 15 min. After centrifugation, the supernatant was analyzed by western blot. Briefly, lysates were subjected to SDS-PAGE and electrotransferred at 100 V for 90 min to PVDF membranes. The membranes were blocked with 5% skim milk for 4 h at room temperature. The membranes were probed with anti-FLAG antibody or anti-ORFV086 polyclonal antiserum, used as the primary antibody, overnight at 4°C. After four washes with TBST (0.1% Tween-20 in TBS), the membranes were incubated for 1 h with a horseradish peroxidase-conjugated goat anti-mouse or goat anti-rabbit antibody. Finally, the chemiluminescent substrates (ECL, Thermo Scientific) were added to develop the immunoblots. Each experiment was repeated at least three times.

### Kinetics of ORFV086 mRNA and protein expression

For RT-PCR analysis, OFTu cells were infected with NA1/11 in the presence or absence of cytosine arabinoside (AraC; 40 μg/ml), an inhibitor of poxvirus late transcription. RNA was extracted at 0, 1, 2, 3, 4, 5, 6, 8, 10, 12, and 24 h post-infection. Specific RT-PCR primers were used to amplify the ORFV086 or ORFV119 gene, depending on the cDNA templates reverse transcribed from the extracted RNA. The primers used for amplifying ORFV086 and ORFV119 were as follows: the forward primer for ORFV086: TTATGACGGCCCCAAACGTGCA; the reverse primer for ORFV086: CCTTACTCACTGTCAAAAGA; The forward primer for ORFV119: TTATGGACTCTCGTAGGC; the reverse primer for ORFV119: TTATCGCTGTCGCTGTCG. To further analyze the kinetics of the ORFV086 protein using western blots, NA1/11 infected OFTu cells were collected simultaneously, as described above. Extracts from infected cells were subjected to SDS-PAGE and immunoblotting using anti-ORFV086 polyclonal antiserum.

### Non-radioactive pulse chase

Click-iT® Metabolic Labeling Reagents for Proteins (Invitrogen) were used in this experiment according to the manufacturer's protocol (Liu et al., 2011). Briefly, OFTu cells were seeded in 100 mm dishes and cultured until 80–90% confluent. The cells were infected with ORFV NA1/11 at an MOI of 10. After washing with warm PBS, cells were starved with methionine-free Dulbecco's modified Eagle's media (Invitrogen), 4 h post-infection, for 1 h. The metabolic labeling reagent Click-iT AHA (50 μM), containing methionine-free media, was then added to infected cells and pulsed for 2 h. Subsequently, the media on the labeled cells was replaced with normal serum MEM media and chased for 17 h. Pulse and chase labeled cells were lysed with 1% SDS in 50 mM Tris-HCl, pH 8.0, containing protease and phosphatase inhibitors, and treated with 250 U/ml Benzonase® endonuclease. Labeled cell lysates were reacted with an alkyne detection molecule which was labeled with biotin using the Click-iT® Protein Reaction Buffer Kit (Invitrogen). Biotin labeled proteins were immunoprecipitated with streptavidin beads and analyzed using western blotting.

## Results

### Kinetic analysis of ORFV086 mRNA and protein expression

**Figure 1:**
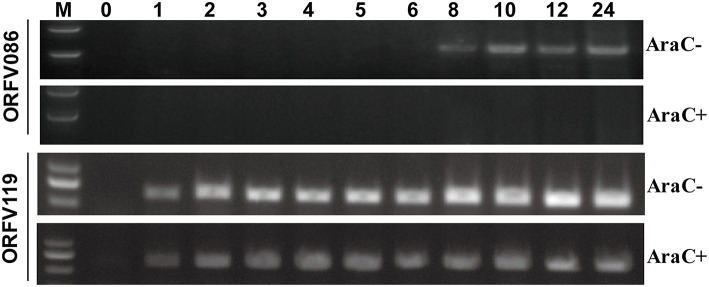
**Kinetic analysis of the ORFV086 protein**. OFTu cells were infected with NA1/11 synchronously in the presence or absence of cytosine arabinoside (AraC; 40 μg/ml) at a multiplicity of infection of 10, and their RNAs were extracted at different times as indicated above. Specific primers were used for RT-PCR of the ORFV086 and the ORFV119 gene, depending on the cDNA templates reverse transcribed by the extracted RNAs. The amplified products were separated in 1.0% agarose gels.

**Figure 2:**
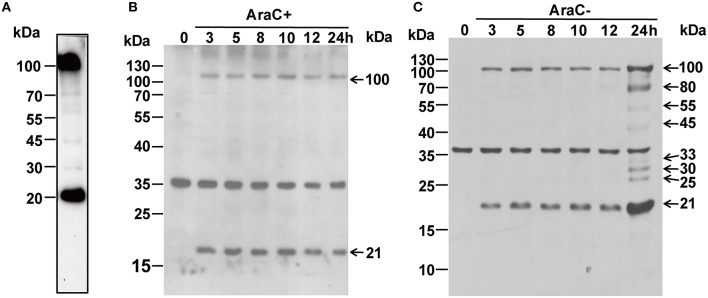
**(A)** Western blot analysis of purified mature ORFV virions using anti ORFV086 pAb. **(B)** Western blot analysis of NA1/11 infected OFTu cells at different time points in the presence of AraC using anti-ORFV086 polyclonal antiserum. **(C)** To determine kinetics of the ORFV086 protein, NA1/11 infected OFTu cells were collected simultaneously at different times as described above. Then, the infected extracts were subjected to SDS-PAGE and immunoblotting using anti-ORFV086 polyclonal antiserum. Positions of the ORFV086 precursor and its derived products were shown with arrows to the right while the molecular weight standards were marked to the left.

Reverse transcription-polymerase chain reaction (RT-PCR) was performed to analyze the kinetics of ORFV086 (Figure 1). The kinetics of ORFV086 gene transcription was compared to that of a known early gene (ORFV119). It was found that ORFV119 gene synthesis started at 1 h post-infection both in the presence and absence of AraC. However, ORFV086 gene expression was detected at 8 h post-infection and could be detected only in those cells cultured in the absence of AraC (Figure 1), indicating that ORFV086 is a late gene. To further analyze the kinetics of the ORFV086 at the protein level, NA1/11 infected OFTu cells were collected and probed with anti-ORFV086 polyclonal antiserum in the absence of AraC (Figure 2C). The expression of ORFV086 started from 12 to 24 h, confirming that ORFV086 expressed at the late stage.

### ORFV086 is a structural protein and is cleaved post ORFV infection

To identify whether or not ORFV086 is a structural protein for the composition of ORFV, western blot was performed using purified mature ORFV virions (Figure 2A). Surprisingly, the ORFV086 full-length protein as well as a peptide of approximately 21 kDa was detected with anti-ORFV086 polyclonal antiserum. When infected with NA1/11, the presence of these two proteins (ORFV086 precursor and 21 kDa fragment) can also be detected after 3 h post infection (p.i.) in the presence of AraC (Figure 2B), indicating that these two proteins are structural proteins for the composition of mature ORFV virions. To verify whether ORFV086 undergoes proteolytic processing in the ORFV viral life cycle, cell extracts taken at different times post-infection, in the absence of AraC, were analyzed with WB using anti-ORFV086 polyclonal antiserum (Figure 2C). WB results showed that, at 3 h post-infection, both the ORFV086 full-length protein and 21 kDa fragment could be detected. Thus, we speculate that the 21 kDa fragment was a product derived from the ORFV086 full-length protein. However, at 24 h post-infection, not only the full length ORFV086 (100 kDa) and the 21 kDa fragment were detected at higher expression levels than at the previous time points, but an additional six fragments of 80, 55, 45, 33, 30, and 25 kDa sizes were detected (Figure 2C). This phenomenon was significantly inhibited by treatment with AraC (Figure 2B). We speculate that these fragments are likely products derived from the ORFV086 protein. Collectively, these results illustrate that ORFV086 and its 21 kDa cleavage fragment are structural proteins of mature ORFV and ORFV086 undergoes proteolytic process.

### ORFV086 protein sequence alignments and phylogenetic analysis

**Figure 3:**
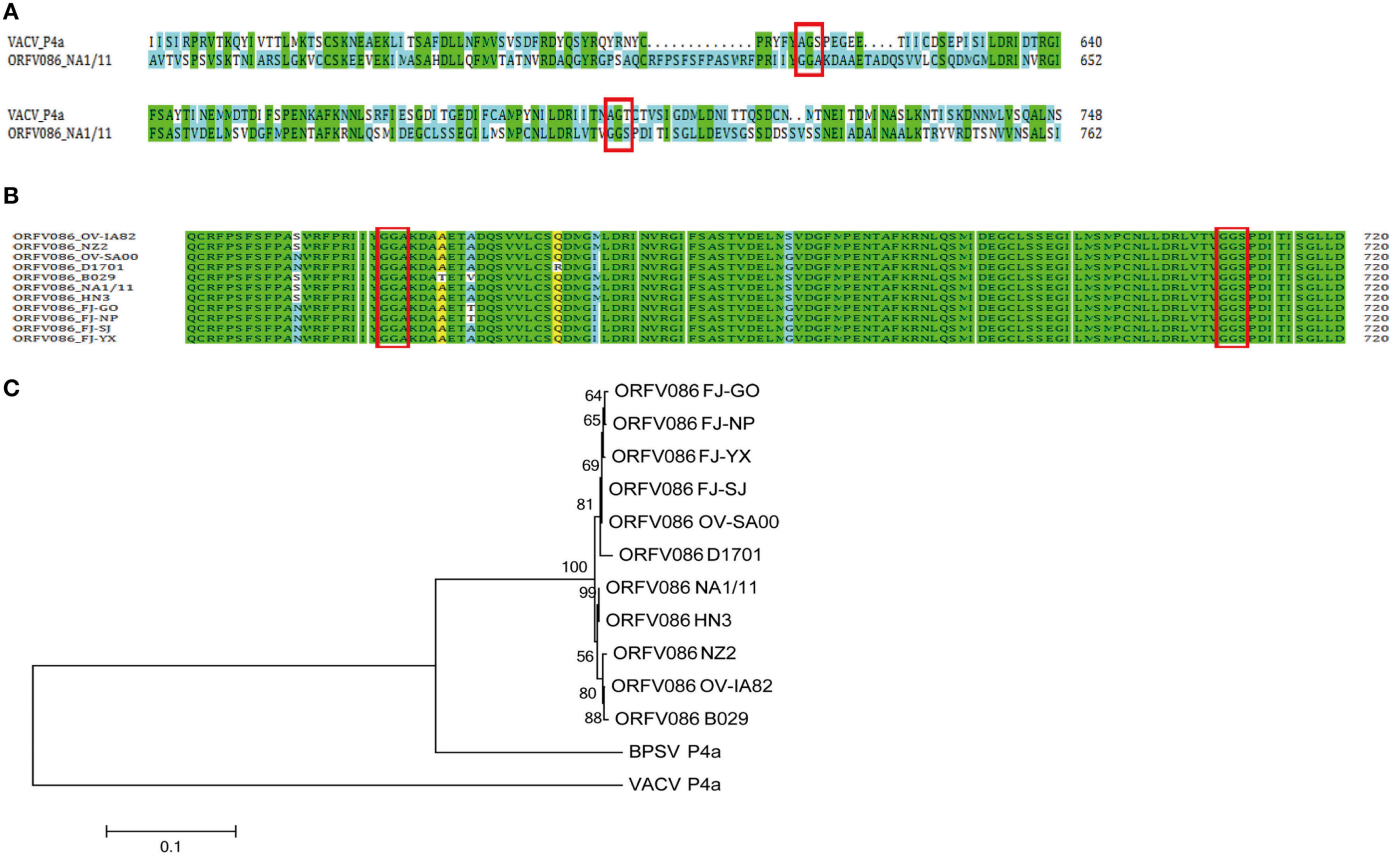
**Sequence alignment and phylogenetic analysis of the ORFV086 protein based on amino acid sequences. (A)** Sequence alignment of the ORFV086 protein of NA1/11 with the P4a protein of VV Copenhagen strain based on their predicted amino acid sequences. The predicted amino acid sequences compared were aligned by using CLUSTAL W. Green color represents 100% identity. Blue color represents no identity. Two putative proteolytic maturation sites are boxed. **(B)** Multiple alignments of corresponding ORFV086 protein among orf virus strains (OV-IA82, NZ2, OV-SA00, D1701, B029, NA1/11, HN3, FJ-GO, FJ-NP, FJ-SJ, and FJ-YX. The predicted amino acid sequences were aligned by using CLUSTAL W. GGA and GGS, two putative proteolytic maturation sites, are boxed. Green color indicates 100% identity. Yellow color represents 80–100% identity. Blue color indicates 50–80% identity. **(C)** Phylogenetic analysis of ORFV086 proteins. The phylogenetic tree constructed using MEGA 5.0 software were generated by orf virus strains OV-IA82, NZ2, OV-SA00, D1701, B029, NA1/11, HN3, FJ-GO, FJ-NP, FJ-SJ, FJ-YX, Vaccinia virus strain(VACV) Copenhagen and Bovine papular stomatitis virus (BPSV) strain BV-AR02.

To further investigate the mechanisms underlying the potential proteolytic sites of ORFV086, the amino acid sequence of the NA1/11 ORFV086 protein were compared with VV Copenhagen strain P4a protein (Figure 3A). It was found that the proteolytic sites of P4a were AGS and AGT, while the predicted corresponding proteolytic sites of ORFV086 were GGA (residues 621-623) and GGS (residues 708-710). Analyzing all ORFV086 proteins in the blast database (Figure 3B), it was found that ORFV086 is a highly conserved structural protein and has a strong similarity among the different strains of ORFV. Additionally, the predicted GGA and GGS proteolytic sites are highly conserved. After further phylogenetic analysis of ORFV086 proteins, VV P4a proteins and BPSV P4a proteins (Figure 3C), the highest homology was found between the NA1/11 and the HN3 strains.

### WB analysis of the recombinant protein with a N-terminal FLAG epitope

**Figure 4:**
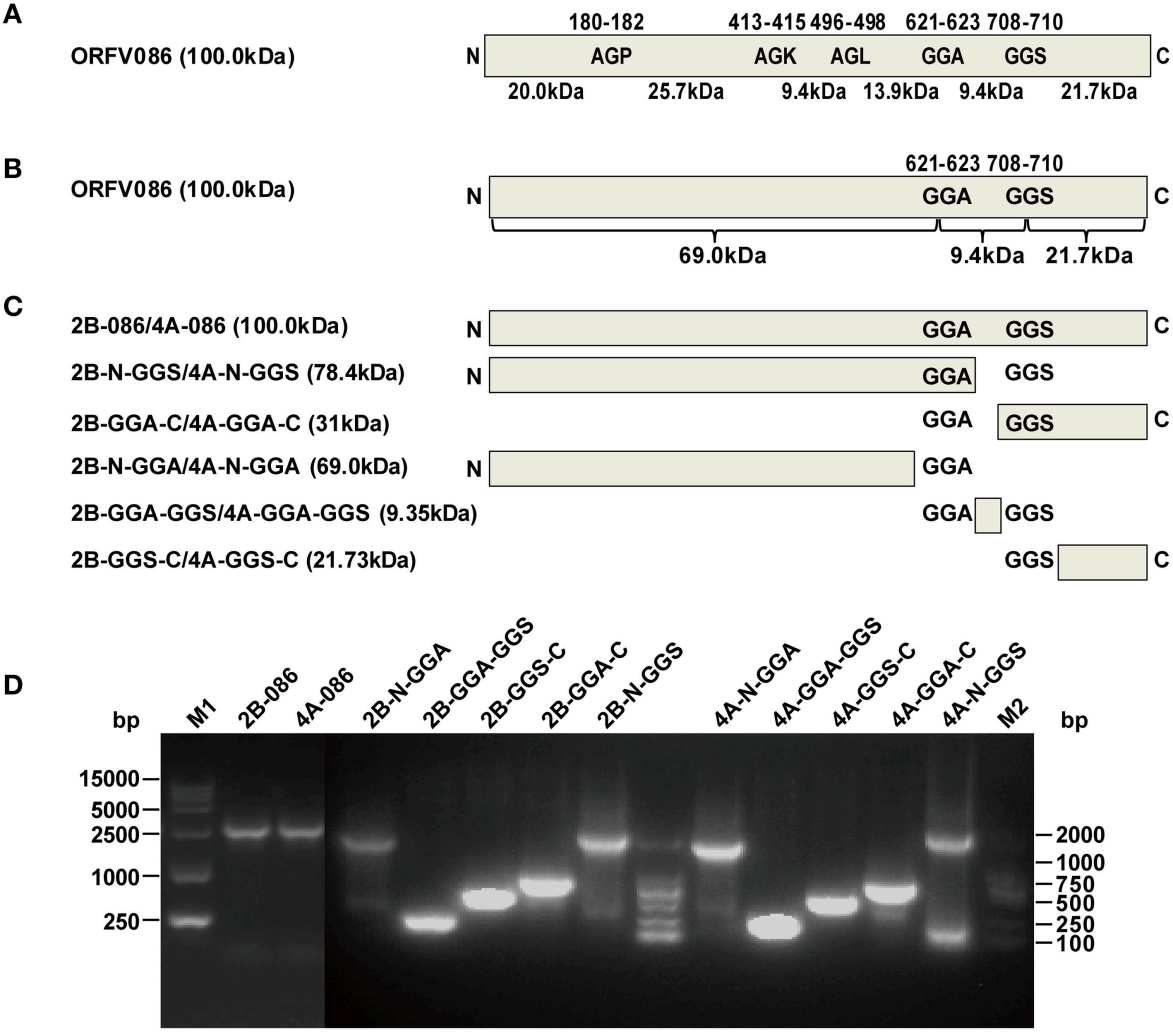
**Design and construction of different ORFV086 fragment expression vector plasmids based on two putative proteolytic maturation sites. (A)** Positions of two putative proteolytic maturation sites and three AGX (AGP, AGK, AGL) motifs in ORFV086 precursor protein. Relative positions of amino acid residues within the precursor are shown by numbers at the top, with predicted molecular weights indicated at the bottom. Schematic cleavage products of all five sites were drawn with molecular weights of 20.0, 25.7, 9.4, 13.9, 9.4, and 21.7 kDa. **(B)** Predicted molecular weights of potential ORFV086 proteolytic products were based on the two putative sites (GGA, GGS). The only utilization of all these two putative proteolytic processing sites (GGA, GGS) would generate a 69.0 kDa N-terminal peptide (residues l-622), a 21.7 kDa C-terminal peptide (residues 710-905) and a small 9.4 kDa internal peptide (residues 623-709). **(C)** Schematic depiction of the construction of different ORFV086 fragment expression vector plasmids. Fragments N-GGA, GGA-GGS, GGS-C were the N-terminus residues (from the N-terminus to the GGA site), the internal residues (from the GGA site to the GGS site) and the C-terminal residues (from the GGS site to the C-terminus) of ORFV086, respectively, cleaving at both GGA and GGS sites. pCMV-Tag2B contained a FLAG epitope at the N-terminus while pCMV-Tag4A contained a FLAG epitope at the C-terminus. **(D)** The results of all ORFV086 fragments amplified by PCR. The PCR products were analyzed in 1.0% agarose gel electrophoresis. Positions and sizes (in bp) were indicated to the left and right.

The NA1/11 ORFV086 gene is 2718 bp, encoding a 100.05 kDa ORFV086 protein. The proteolytic sites of VV P4a are AGS and AGT, while the corresponding ORFV086 predicted proteolytic sites are GGA (residues 708-710) and GGS (residues 621-623) (Figure 4A). In addition to GGA and GGS, AGP (residues 180-182), AGK (residues 413-415), and AGL (residues 496-498) were also found, similar to the AGX motif in the proteolytic sites of the VV core protein (Byrd and Hruby, 2006). They are likely to be the proteolytic maturation sites of ORFV086. The location of five sites (GGA, GGS, AGP, AGK, and AGL) in the ORFV086 protein and the molecular mass of the peptides are shown in Figure 4A.

To facilitate the study of ORFV086 processing, we focused on whether or not GGA and GGS are proteolytic sites. The ORFV086 precursor protein is divided into an N-terminus of 69 kDa, a C-terminus of 21.7 kDa and a middle fragment of 9.4 kDa, bounded by GGA and GGS (Figure 4B). Using the NA1/11 strain genome as a template, we successfully produced six pairs of eukaryotic expression vectors by cloning the full-length ORFV086 and different deletion mutants into pCMV-Tag2B and pCMV-Tag4A respectively after PCR amplification and DNA sequencing (Figures 4C,D).

**Figure 5:**
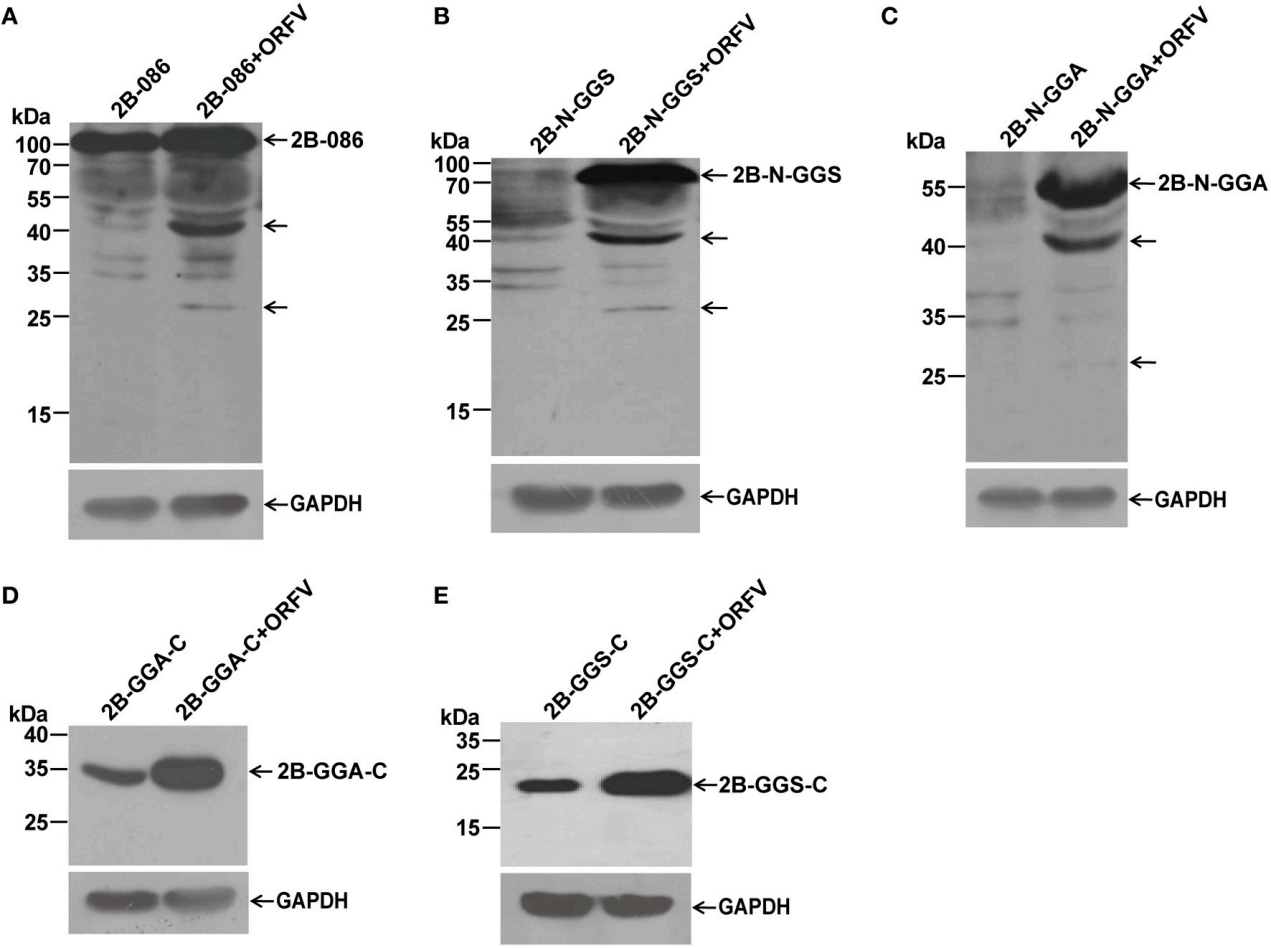
**Immunoblot detection of proteolytic processing of different ORFV086 fragment proteins with an N-terminal FLAG epitope by using anti-FLAG mAb**. OFTu cells were transfected with 2B-086, 2B-N-GGS, 2B-N-GGA, 2B-GGA-C, and 2B-GGS-C, following infection or lack of infection with ORFV NA1/11. After transfection for 24 h, cells were harvested and subjected to SDS-PAGE and immunoblotting using the anti-FLAG monoclonal antibody. 2B-086, 2B-N-GGS, 2B-N-GGA, 2B-GGA-C, and 2B-GGS-C in **(A–E)** were transfected with the corresponding expression plasmids, while 2B-086+ORFV, 2B-N-GGS+ORFV, 2B-N-GGA+ORFV, 2B-GGA-C+ORFV, and 2B-GGS-C+ORFV in **(A–E)** were transfected with the corresponding expression plasmids and synchronously infected with ORFV NA1/11. The expression of GAPDH served as a loading control. Positions of different ORFV086 fragment proteins and their derivatives were shown with arrows to the right while the molecular weight standards were marked to the left.

Cell extracts of the infected and uninfected groups were used for western blotting, using anti-FLAG mAb in order to determine the location of the ORFV086 proteolytic site (Figure 5).

Comparing pCMV-Tag2B-086 (2B-086) + ORFV with 2B-086, pCMV-Tag2B-N-GGS (2B-N-GGS) + ORFV with 2B-N-GGS, and pCMV-Tag2B-N-GGA (2B-N-GGA) + ORFV with 2B-N–GGA through the analysis of anti-FLAG mAb, there were two specific bands of 45 kDa and 25 kDa (Shown by the arrows in Figures 5A–C). Since there was a FLAG-tag attached in the N-terminus, we can infer that the proteolytic sites of the 45 kDa and 25 kDa fragments are located in the N-GGA fragment (69 kDa in size). When comparing pCMV-Tag2B-GGA-C (2B-GGA-C) + ORFV with 2B-GGA-C and pCMV-Tag2B-GGS-C (2B-GGS-C) + ORFV with 2B-GGS-C, we did not find bands other than the target bands, although we expected a 9 kDa band at 2B-GGA-C + ORFV (Figures 5D,E). Possible reason for the missing small fragments is that FLAG-tagged small peptides would be degraded when the cleavage occurred in the neighborhood of the C- or N- terminus or the FLAG-tag hampered protein folding, resulting in only partial cleavage.

### WB analysis of the recombinant protein with a C-terminal FLAG epitope

**Figure 6:**
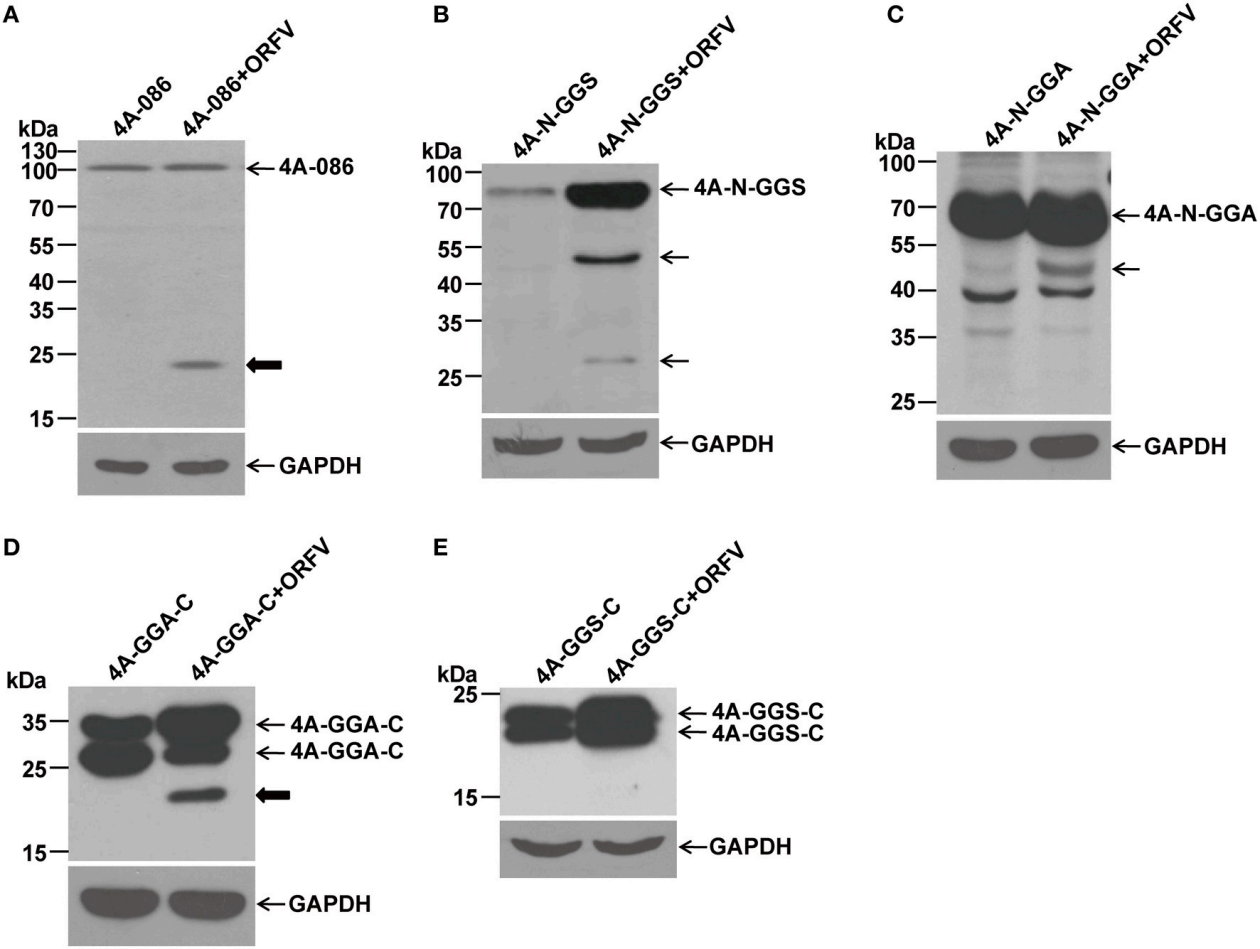
**Immunoblotting of the proteolytic maturation of different ORFV086 fragment proteins with a C-terminal FLAG epitope using anti-FLAG mAb**. OFTu cells were transfected with 4A-086, 4A-N-GGS, 4A-N-GGA, 4A-GGA-C, and 4A-GGS-C, following infection or lack of infection with ORFV NA1/11. After 24 h incubation, cells were harvested and subjected to SDS-PAGE and immunoblotting using anti-FLAG mAb. 4A-086, 4A-N-GGS, 4A-N-GGA, 4A-GGA-C, and 4A-GGS-C in **(A–E)** were transfected only with corresponding expression plasmids, while 4A-086+ORFV, 4A-N-GGS+ORFV, 4A-N-GGA+ORFV, 4A-GGA-C+ORFV, and 4A-GGS-C+ORFV in **(A–E)** were transfected with the corresponding expression plasmids and infected with ORFV NA1/11. The expression of GAPDH served as a loading control. Positions of different ORFV086 fragment proteins and their derivatives were shown with arrows to the right. Thick arrows indicate the 21 kDa cleavage fragment.

Recombinant plasmids pCMV-Tag4A-086 (4A-086), pCMV-Tag4A-N-GGS (4A-N-GGS), pCMV-Tag4A-GGA-C (4A-GGA-C), pCMV-Tag4A-N-GGA (4A-N-GGA), pCMV-Tag4A-GGA-GGS (4A-GGA-GGS), and pCMV-Tag4A-GGS-C (4A-GGS-C), with the FLAG-tag in the C terminus, were transfected into OFTu cells, both infected and uninfected with ORFV NA1/11. We compared the proteolytic differences between the groups with or without the virus. Western blotting was performed as described using the N-terminal anti-FLAG mAb (Figure 6).

By analyzing with anti-FLAG mAb (Figure 6), it was found that a specific peptide of 23 kDa could be detected in 4A-086+ ORFV (Figure 6A) and 4A-GGA-C+ ORFV (Figure 6D). Because there is a FLAG-tag attached to its C-terminus, we speculate that the 23 kDa band is the 4A-GGS-C protein (about 23 kDa, namely 21.7 kDa of GGS-C plus 1 kDa of FLAG-tag) proteolytically processed at the GGS site. Comparing 4A-N-GGS + ORFV with 4A-N-GGS and 4A-N-GGA + ORFV with 4A-N-GGA, there was a specific band of 45 kDa (Figures 6B,C). Because there is a FLAG-tag at its C-terminus, we speculate that the proteolytic site of the 45 kDa band is located in the N-GGA fragment (69 kDa in size). Comparing this to 4A-GGS-C, there were no other bands in addition to the target protein in 4A-GGS-C + ORFV (e.g., Figure 6E).

### WB analysis of the full-length mutant vectors of ORFV086

**Figure 7:**
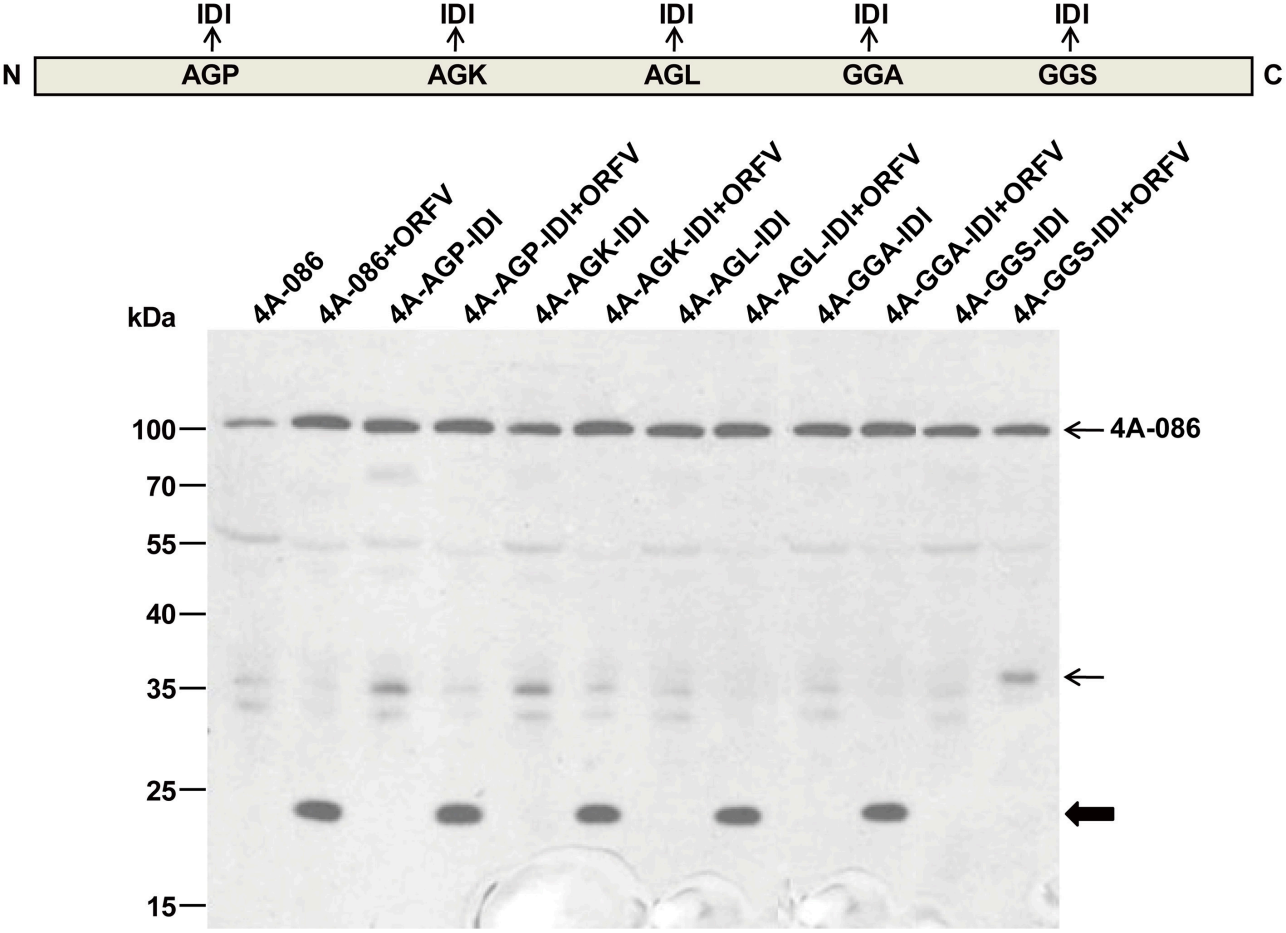
**Immunoblotting of the genetic mutation of ORFV086 precursor proteins with a C-terminal FLAG epitope using anti-FLAG mAb**. OFTu cells were transfected with those five mutants of 4A-086 (lanes 3–12), following infection or lack of infection with ORFV NA1/11. 4A-086, 4A-AGP-IDI, 4A-AGK-IDI, 4A-AGL-IDI, 4A-GGA-IDI, and 4A-GGS-IDI were transfected with only the corresponding expression plasmid, while 4A-086+ORFV, 4A-AGP-IDI+ORFV, 4A-AGK-IDI+ORFV, 4A-AGL-IDI+ORFV, 4A-GGS-IDI+ORFV, and 4A-GGS-IDI+ORFV were transfected and infected simultaneously. Positions of ORFV086 precursor and their derivatives were marked with arrows to the right.

To determine whether the 23 kDa fragment (Figure 6, thick arrow) is a product derived from ORFV086, the AGP, AGK, AGL, GGA, and GGS sites of full length 4A-086 were mutated to IDI, transfected into OFTu cells and the differences in immunoblot bands between the infected and uninfected groups were compared. Analysis using anti-FLAG mAb found that, after infection with ORFV virus, the mutant 4A-GGS-IDI + ORFV (lane 12), with the GGS site mutated to IDI, did not display the 23 kDa band (thick arrow) (Figure 7). Mutants 4A-AGP-IDI + ORFV, 4A-AGK-IDI + ORFV, 4A-AGL-IDI + ORFV, and 4A-GGA-IDI + ORFV (lanes 4, 6, 8, 10) showed a 23 kDa protein, similarly with the non-mutated 4A-086 + ORFV (lane 2; shown with thick arrow in Figure 7). This suggests that the 23 kDa band is 4A-GGS-C, which cleaves at the GGS site of the ORFV086 precursor.

**Figure 8:**
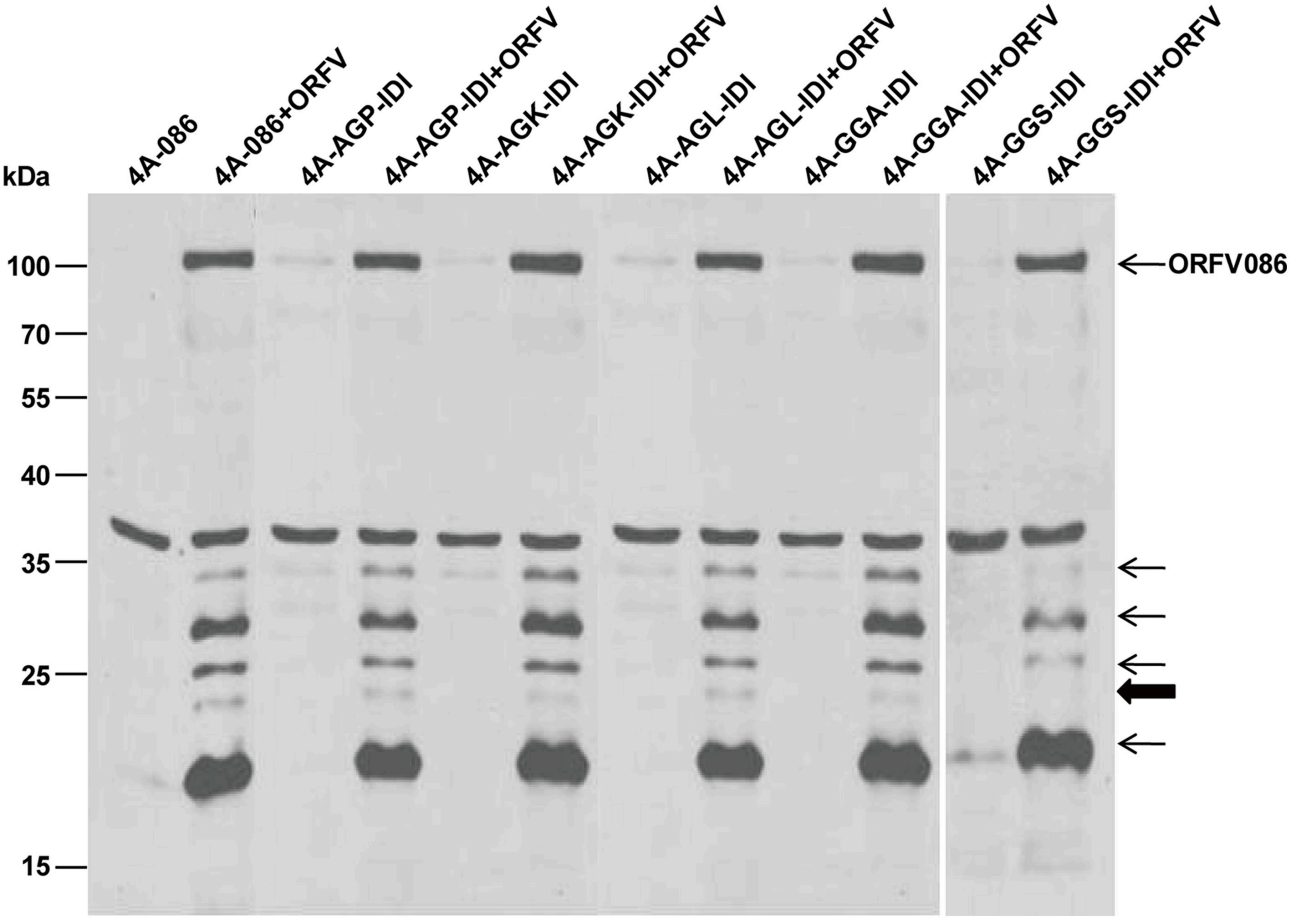
**Genetic mutation of ORFV086 precursor proteins with a C-terminal FLAG epitope**. OFTu cells were transfected with five mutants of 4A-086 [AGX (AGP, AGK, AGL) and GGA, GGS] (lanes 3–12), following infection or no infection with ORFV NA1/11. 4A-086, 4A-AGP-IDI, 4A-AGK-IDI, 4A-AGL-IDI, 4A-GGA-IDI, and 4A-GGS-IDI were transfected with corresponding expression plasmids alone, while 4A-086+ORFV, 4A-AGP-IDI+ORFV, 4A-AGK-IDI+ORFV, 4A-AGL-IDI+ORFV, 4A-GGS-IDI+ORFV, and 4A-GGS-IDI+ORFV were transfected and infected. The extracts of infected lines were separated by SDS-PAGE and immunoblotting using anti-ORFV086 polyclonal antiserum. Positions of proteins which were identified specifically by anti-ORFV086 polyclonal antiserum were marked with arrows to the right.

Similarly, WB analysis using anti-ORFV086 polyclonal antiserum (Figure 8) found that the 23 kDa band could be detected in 4A-AGP-IDI + ORFV, 4A-AGK-IDI + ORFV, 4A-AGL-IDI + ORFV, 4A-GGA-IDI + ORFV (lanes 4, 6, 8, 10), and 4A-086 + ORFV (lane 2), but not in 4A-GGS-IDI + ORFV (lane 12) with the GGS mutated to IDI (shown in bold arrow in Figure 8). This indicates that the 23 kDa bands (thick arrow shown) is 4A-GGS-C which cleaves at the GGS site of ORFV086. These results further demonstrate that proteolysis of ORFV086 occurs at the GGS site.

### Pulse-chase

**Figure 9:**
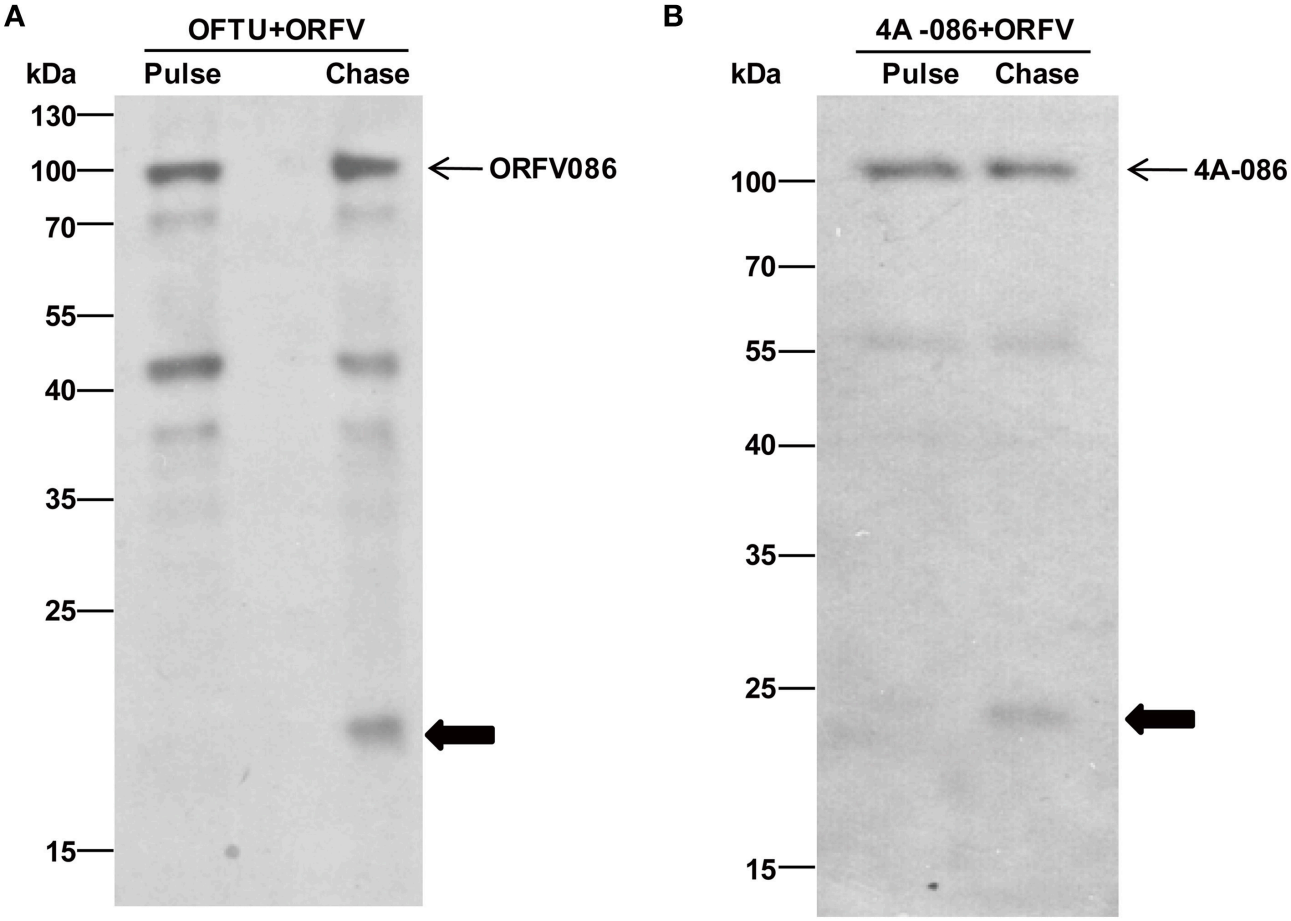
**Immunoblotting of pulse-chase labeled ORFV086 protein using the anti-ORFV086 polyclonal antiserum or anti-FLAG mAb. (A)** Pulse chase of the ORFV086 protein. Cells were lysed, labeled with biotinylated alkyne and analyzed via WB using anti-ORFV086 antiserum. Positions of the ORFV086 precursor and their derivatives are marked with arrows to the right. **(B)** Pulse-chase of the 4A-086 protein. Pulse and chase labeled cells were lysed, labeled with biotinylated alkyne and analyzed via WB using anti-FLAG mAb. Positions of 4A-086 precursor and their derivatives were marked with arrows to the right.

A pulse-chase assay was performed to trace the biotinylated synthesized substrate. The biotinylated protein of the pulse-chase experiment was immunoprecipitated with streptavidin beads and analyzed by western blotting using anti-ORFV086 polyclonal antiserum. The results show (Figure 9A) that the 21 kDa product is a proteolytic protein of ORFV086. The ORFV086 precursor could not be detected in OFTu cells infected with ORFV after 3 h. This further illustrates ORFV086 is a late gene.

Likewise, in order to prove that the 23 kDa product is indeed the product of proteolysis of the full-length 4A-086 protein, non-radioactive pulse-chase analysis of OFTu cells was used after transfection with 4A-086 (Figure 9B). We continued to use conventional serum media to chase 24 h after infection with NA1/11. The results show that the 23 kDa product is chased from the 4A-086 protein (Figure 9B). This also confirms that the proteolytic product of 4A-GGS-C is a 23 kDa protein.

Notably, the ORFV086 precursor (Figure 9A) and 4A-086 full-length protein (Figure 9B) were also detected in non-radioactive chase-labeled proteins, suggesting that the proteolytic processing of the ORFV086 and 4A-086 proteins did not proceed to completion. This is consistent with the previous hypothesis that the ORFV086 full-length protein and 21 kDa cleavage fragment are structural proteins which are assembled to compose mature ORFV virions.

## Discussion

### Late gene

RT-PCR kinetic analysis revealed the synthesis of ORFV086 could be detected 8 h after infection with NA1/11, and could be detected only in those cells cultured in the absence of AraC, an inhibitor of late poxvirus transcription. However, ORFV119 gene synthesis started at 1 h post-infection both in the presence and in the absence of AraC, indicating that ORFV086 gene is a late gene. Using western blot, ORFV086 full-length protein, a 21 kDa fragment and other derivatives could be detected with a higher expression in the first 24 h, indicating that newly synthesized ORFV086 started to appear between 12 and 24 h post infection. Further pulse-chase experiments showed that the synthesis of the ORFV086 protein could be detected 5 h after infection with NA1/11. These results demonstrate that ORFV086 is a late gene.

### Structural proteins

Here our results evidenced that the ORFV086 full-length protein and the 21 kDa fragment are structural proteins composing ORFV mature virions. Both ORFV086 precursor and the 21 kDa fragment were directly observed in mature ORFV virions. In cases of infection with ORFV, anti-ORFV086 polyclonal antiserum can be used to detect the presence of these two proteins, either with or without treatment with AraC. Pulse-chase experiments found that the ORFV086 protein undergoes incomplete proteolysis, in contrast to the complete proteolysis of the VV P4a protein. However, this was unlike the proteolysis of the VV P4a precursor, in which the 21kDa protein is a cleaved fragment but not a structural component of virus particles (Heljasvaara et al., 2001; Yang, 2007). In addition to the 21 kDa protein and the ORFV086 precursor, anti-ORFV086 polyclonal antiserum can also detect multiple bands, likely proteolytic products of ORFV086 or viral protein cross-reactions with polyclonal antiserum.

### GGS proteolytic site

Although, there are five potential proteolytic sites, including three AGX motifs (AGP, AGK, and AGL) and two GGX motifs (GGA, GGS) located in ORFV086 protein, only the GGS site was verified experimentally. In experiments of transient expression of recombinant proteins fused with a C-terminal FLAG epitope and genetic mutations of the ORFV086 precursor, the only certainty is that the 23 kDa product is 4A-GGS-C derived from the 4A-086 precursor cleaved at the GGS site which was further confirmed in the pulse-chase assay. Thus, the 4A-086 protein is proteolytically processed at GGS, which is different from the AGX motif in cleaving the VV P4a protein, but similar to the GGX motif of adenoviruses and the African swine fever virus (López-Otín et al., 1989). A possible explanation is that the proteolytic maturation pathway utilized to process the ORFV086 protein is different from that of the VV P4a protein.

### The 21 kDa fragment is a product cleaved at GGS site

Pulse-chase experiments showed that a 21 kDa protein was chased from ORFV086 while a 23 kDa protein was chased from 4A-086. The 23 kDa product is 4A-GGS-C derived from the 4A-086 precursor cleaved at the GGS site. This suggests that GGS is the proteolytic site of ORFV086. We can infer that the 21 kDa ORFV086 product is a GGS-C fragment derived from the ORFV086 precursor cleaved at GGS site. Therefore, the proteolysis of ORFV086 protein happens at the GGS site to produce GGS-C of 21 kDa. In addition to the GGS site, other proteolytic sites were present in full length ORFV086, evidenced by the presence of seven bands, apart from ORFV086 precursor, detected by anti ORFV086 pAb 24 h post infection. According to our results, we suspect that three proteolytic sites may be located in the N-GGA fragment. One site resulted in a 40 kDa band starting from the N-terminus of ORFV086 (Figures 4A–C). The second site produced a 45 kDa band ending at the GGS site (Figure 5B). The third site generated another 45 kDa band ending at the GGA site (Figure 5C). Further, studies are required to identify where, exactly, these specific proteolytic sites are located, and how these sites function.

## Conclusion

In summary, ORFV086 is a late gene. Proteolytic maturation of ORFV086 at the GGS site does not proceed to completion and forms the GGS-C product. Both the ORFV086 precursor and 21 kDa fragment (GGS-C) are structural proteins composing mature ORFV virions, which is in stark contrast to the VV P4a protein. In addition to the GGS site, there may be other proteolytic sites in the N-GGA fragment of ORFV086. Overall, this research provides new insight into viral cleavage processing and enlarges the understanding of the biological function of ORFV086. The identification of the GGS cleavage site in ORFV086 revealed a novel clinical target for drugs designed against ORF virus assembly.

## Author contributions

SL, WH, and ML participated in design of the study. XW, BX, JZ, DC performed the experiments. XW, BX, WL analyzed the data and wrote the manuscript. All authors read and approved the final manuscript.

## Funding

This study was supported by grants (No. 31070138 and No. 31170147) from the National Natural Science Foundation of China (NSFC) and Special Funds for Colleges and Universities Talents by Guangdong Province (2011) and Scientific Research Foundation of Introducing Talents of Southern Medical University (2010). The funders had no role in study design, data collection and analysis, decision to publish or preparation of the manuscript.

### Conflict of interest statement

The authors declare that the research was conducted in the absence of any commercial or financial relationships that could be construed as a potential conflict of interest.

## References

[B1] Byrd C. M. Hruby D. E. (2006). Vaccinia virus proteolysis–a review. Rev. Med. Virol. 16, 187–202. 10.1002/rmv.499 16710840 PMC7169229

[B2] Chi X. Zeng X. Hao W. Li M. Li W. Huang X.. (2013). Heterogeneity among orf virus isolates from goats in Fujian Province, Southern China. PLoS ONE 8:e66958. 10.1371/journal.pone.0066958 24143166 PMC3797069

[B3] Cottone R. Büttner M. Bauer B. Henkel M. Hettich E. Rziha H. J. (1998). Analysis of genomic rearrangement and subsequent gene deletion of the attenuated Orf virus strain D1701. Virus Res. 56, 53–67. 10.1016/S0168-1702(98)00056-2 9784065

[B4] Delhon G. Tulman E. R. Afonso C. L. Lu Z. de la Concha-Bermejillo A. Lehmkuhl H. D.. (2004). Genomes of the parapoxviruses ORF virus and bovine papular stomatitis virus. J. Virol. 78, 168–177. 10.1128/JVI.78.1.168-177.2004 14671098 PMC303426

[B5] Diel D. G. Luo S. Delhon G. Peng Y. Flores E. F. Rock D. L. (2011). A nuclear inhibitor of NF-kappaB encoded by a poxvirus. J. Virol. 85, 264–275. 10.1128/JVI.01149-10 20980501 PMC3014193

[B6] Heljasvaara R. Rodríguez D. Risco C. Carrascosa J. L. Esteban M. Rodríguez J. R. (2001). The major core protein P4a (A10L gene) of vaccinia virus is essential for correct assembly of viral DNA into the nucleoprotein complex to form immature viral particles. J. Virol. 75, 5778–5795. 10.1128/JVI.75.13.5778-5795.2001 11390580 PMC114294

[B7] Hellen C. U. Wimmer E. (1992a). Maturation of poliovirus capsid proteins. Virology 187, 391–397. 10.1016/0042-6822(92)90440-Z 1312265

[B8] Hellen C. U. Wimmer E. (1992b). The role of proteolytic processing in the morphogenesis of virus particles. Experientia 48, 201–215. 10.1007/BF01923512 1740191 PMC7087542

[B9] Joklik W. K. (1962). The purification fo four strains of poxvirus. Virology 18, 9–18. 10.1016/0042-6822(62)90172-1 14036977

[B10] Lee P. Hruby D. E. (1993). trans processing of vaccinia virus core proteins. J. Virol. 67, 4252–4263. 7685413 10.1128/jvi.67.7.4252-4263.1993 PMC237795

[B11] Li H. Ning Z. Hao W. Zhang S. Liao X. Li M.. (2012). Identification and characterization of monoclonal antibodies against the ORFV059 protein encoded by Orf virus. Virus Genes 44, 429–440. 10.1007/s11262-011-0710-9 22237464

[B12] Li W. Ning Z. Hao W. Song D. Gao F. Zhao K.. (2012). Isolation and phylogenetic analysis of orf virus from the sheep herd outbreak in northeast China. BMC Vet. Res. 8:229. 10.1186/1746-6148-8-229 23174032 PMC3561078

[B13] Liu K. Yang P. Y. Na Z. Yao S. Q. (2011). Dynamic monitoring of newly synthesized proteomes: up-regulation of myristoylated protein kinase A during butyric acid induced apoptosis. Angew. Chem. Int. Ed Engl. 50, 6776–6781. 10.1002/anie.201102542 21678537

[B14] Lojkic I. Cac Z. Beck A. Bedekovic T. Cvetnic Z. Sostaric B. (2010). Phylogenetic analysis of Croatian orf viruses isolated from sheep and goats. Virol. J. 7:314. 10.1186/1743-422X-7-314 21073725 PMC2989325

[B15] López-Otín C. Símon-Mateo C. Martínez L. Viñuela E. (1989). Gly-Gly-X, a novel consensus sequence for the proteolytic processing of viral and cellular proteins. J. Biol. Chem. 264, 9107–9110. 2722819

[B16] Mercer A. A. Fraser K. Barns G. Robinson A. J. (1987). The structure and cloning of orf virus DNA. Virology 157, 1–12. 10.1016/0042-6822(87)90307-2 3029950

[B17] Mercer A. A. Ueda N. Friederichs S. M. Hofmann K. Fraser K. M. Bateman T.. (2006). Comparative analysis of genome sequences of three isolates of Orf virus reveals unexpected sequence variation. Virus Res. 116, 146–158. 10.1016/j.virusres.2005.09.011 16274827

[B18] Rodriguez D. Bárcena M. Möbius W. Schleich S. Esteban M. Geerts W. J.. (2006). A vaccinia virus lacking A10L: viral core proteins accumulate on structures derived from the endoplasmic reticulum. Cell. Microbiol. 8, 427–437. 10.1111/j.1462-5822.2005.00632.x 16469055

[B19] Saitou N. Nei M. (1987). The neighbor-joining method: a new method for reconstructing phylogenetic trees. Mol. Biol. Evol. 4, 406–425. 3447015 10.1093/oxfordjournals.molbev.a040454

[B20] Tamura K. Peterson D. Peterson N. Stecher G. Nei M. Kumar S. (2011). MEGA5: molecular evolutionary genetics analysis using maximum likelihood, evolutionary distance, and maximum parsimony methods. Mol. Biol. Evol. 28, 2731–2739. 10.1093/molbev/msr121 21546353 PMC3203626

[B21] Tan J. L. Ueda N. Mercer A. A. Fleming S. B. (2009). Investigation of orf virus structure and morphogenesis using recombinants expressing FLAG-tagged envelope structural proteins: evidence for wrapped virus particles and egress from infected cells. J. Gen. Virol. 90, 614–625. 10.1099/vir.0.005488-0 19218206

[B22] VanSlyke J. K. Franke C. A. Hruby D. E. (1991). Proteolytic maturation of vaccinia virus core proteins: identification of a conserved motif at the N termini of the 4b and 25K virion proteins. J. Gen. Virol. 72 (Pt 2), 411–416. 10.1099/0022-1317-72-2-411 1993877

[B23] Vanslyke J. K. Whitehead S. S. Wilson E. M. Hruby D. E. (1991). The multistep proteolytic maturation pathway utilized by vaccinia virus P4a protein: a degenerate conserved cleavage motif within core proteins. Virology 183, 467–478. 10.1016/0042-6822(91)90976-I 1853556

[B24] Whitehead S. S. Hruby D. E. (1994). Differential utilization of a conserved motif for the proteolytic maturation of vaccinia virus proteins. Virology 200, 154–161. 10.1006/viro.1994.1174 8128619

[B25] Xiao B. Kuang Z. Zhan Y. Chen D. Gao Y. Li M.. (2016). A Novel Polyclonal Antiserum against Toxoplasma gondii Sodium Hydrogen Exchanger 1. Korean J Parasitol. 54, 21–29. 10.3347/kjp.2016.54.1.21 26951975 PMC4792324

[B26] Yang S. J. (2007). Characterization of vaccinia virus A12L protein proteolysis and its participation in virus assembly. Virol. J. 4:78. 10.1186/1743-422x-4-78 17678539 PMC1959187

